# Association between smoking and the peripheral vestibular disorder: a retrospective cohort study

**DOI:** 10.1038/s41598-017-17294-1

**Published:** 2017-12-04

**Authors:** Masaoki Wada, Taro Takeshima, Yosikazu Nakamura, Shoichiro Nagasaka, Toyomi Kamesaki, Eiji Kajii, Kazuhiko Kotani

**Affiliations:** 1 Division of Community and Family Medicine, Center for Community Medicine, Jichi Medical University, Shimotsuke, Japan; 2 Oki Clinic, Yuki, Japan; 3 Department of Public Health, Jichi Medical University, Shimotsuke, Japan; 4 Division of Diabetes, Metabolism and Endocrinology, Showa University Fujigaoka Hospital, Yokohama, Japan

## Abstract

Common inner ear diseases include peripheral vestibular disorder (PVD) and hearing impairment. The association between smoking and peripheral vestibular disorder (PVD) is unclear. We examined associations between smoking and new PVD events. In this retrospective study, we consecutively enrolled 393 participants aged ≥20 years [mean age 65.3 years; males 133 (33.8%)] treated for hypertension, dyslipidaemia, or diabetes mellitus at a primary care clinic between November 2011 and March 2013. Participants were categorized as ever-smokers (including current and past -smokers; divided per <30 and ≥30 pack-years), and never-smokers. New PVD events were reported over a 1-year follow-up period. Hazard ratios (HR) for new onset PVD were estimated using the Cox proportional hazard regression model. Compared to never-smokers, the adjusted HR was 2.22 for ever-smokers and 2.70 for all ever-smokers with ≥30 pack-years among all 393 participants. Among male participants, compared to never-smokers, the adjusted HR was 4.41 for ever-smokers with ≥30 pack-years. A smoking history of ≥30 pack-years was strongly associated with the risk of new onset PVD in males but not, females. This study may assist patients with smoking cessation for the prevention of new PVD events among males.

## Introduction

Epidemiological and clinical studies indicate that smoking is a risk factor for cardiovascular and cerebrovascular disease, cancer, and a range of other serious illnesses^1–3^. Common inner ear diseases include peripheral vestibular disorder (PVD) and hearing impairment^4^. The association between sensorineural hearing impairment and smoking has been reported previously^5–8^. The Korea National Health and Nutrition Examination Survey found that current smoking was associated with hearing impairment in both speech-band and high frequencies, across all ages^5^. The Epidemiology of Hearing Loss Study reported that current smoking was associated with an increased risk of hearing impairment^6^. Within a Bangladeshi population, smokers had significantly higher hearing thresholds at 4, 8, and 12 kHz frequencies than non-smokers^7^. Current-smokers are more likely to experience hearing loss than non-smokers^8^.

The mechanisms that underlie dizziness/vertigo are unclear. PVD encompasses a group of diseases including vestibular neuritis (VN), benign paroxysmal positional vertigo (BPPV), and Meniere’s disease (MD); however, its mechanisms of onset are unclear^9,10^. Previous studies that examined associations between PVD and smoking produced inconsistent findings. Vertigo treatments may be ineffective with smokers^11^. Another study reported that smokers tended to recover earlier from BPPV after approximately 19 days^12^. Some recent reports suggest an association between atherosclerosis and PVD. Smoking, the established risk factor for the atherosclerosis, may act both directly and indirectly on feeding vessels and cause vertigo^13,14^.

Despite a previous review by our research group^14,15^, it is unclear if cigarette smoking is associated with PVD. We conducted this study to determine if an association exists between cigarette smoking and new PVD events. An association between smoking and PVD events might prompt patients with PVD to more aggressively pursue smoking cessation treatment, potentially preventing new PVD events and smoking-related diseases.

## Methods

This study was approved by the clinical research Institutional Review Board of Jichi Medical University (approval number: 14-01; approval date: May 22, 2014). According to the ethical guidelines for Medical and Health Research Involving Human Subjects^16^, in this research design, written informed consent is not necessarily required. Therefore, we explained the outline of our study and provided opportunities for disagreement. All participants consented to involvements in this study.

All study procedures were carried out in accordance with the STROBE statement^17^. All the examinations and medical reviews involved in this study are regularly performed, standard-of-care procedures for participants with hypertension, dyslipidaemia, or diabetes mellitus regardless of enrollment; hence, there were no additional study-related procedures or risks to the participants. We noted participants’ consent in their medical records.

### Study design, participants, and setting

This was a retrospective cohort study. We consecutively enrolled 349 participants who were treated for hypertension, dyslipidaemia, or diabetes mellitus, for at least 6 months at a single primary care clinic (Oki Clinic) between November 2011 and March 2013. All the participants were ≥20 years old, and were followed for at least one year; we examined all participants for signs and symptoms of PVD. We have previously described the Oki Clinic^15^.

Variables of interest included age, sex, smoking status, alcohol consumption status, systolic blood pressure (mmHg), diastolic blood pressure (mmHg), total cholesterol (TC, mg/dL), low-density lipoprotein cholesterol (LDL-C, mg/dL), high-density lipoprotein cholesterol (HDL-C, mg/dL), triglycerides (TG, mg/dL), and glycosylated haemoglobin [HbA1c, NGSP (National Hemoglobin Standardization Program); %], as baseline data. Smoking status was categorized as current, past, and never smoker; a current smoker was defined as a person who smoked at least one cigarette daily by the time of baseline data acquisition, a past smoker was someone who quit smoking by the time of baseline data acquisition, but who had smoked previously.

Alcohol consumption status was categorized as current, former, and never drinker; a current drinker was defined as a person who drank in the 12 months prior to baseline data acquisition, a former drinker was someone who quit drinking by the time of baseline data acquisition, but drank previously. Dyslipidaemia was defined as LDL-C ≥130 mg/dL, HDL-C <40 mg/dL in men and <50 mg/dL in women, TG ≥150 mg/dL, TC ≥200 mg/dL, or taking medication for dyslipidaemia^18^. Hypertension was defined as systolic blood pressure ≥140 mmHg, diastolic blood pressure ≥90 mmHg, or the use of anti-hypertensive medication^19^. Diabetes mellitus was defined as fasting plasma glucose ≥126 mg/dL, non-fasting plasma glucose ≥200 mg/dL, HbA1c (NGSP) ≥6.5%, or the use of anti-hyperglycaemic medication^20^.

### Smoking status and smoking consumption (pack-years)

We classified smoking status as non-smoker and ever-smoker. We combined past-smokers and current-smokers into an ever-smoker group, and classified smoking status as never-smoker or ever-smoker for comparison, referring to previous studies^21,22^. Ever-smokers were further divided into two subgroups, using the cut-off value of 30 pack-years, based on previous studies, because a smoking consumption of over 30 pack-years is a well-known established risk factor for lung cancer, chronic obstructive pulmonary disease (COPD), and cardiovascular diseases^23–26^. We calculated pack-years as the number of cigarettes packs per day × years of smoking (one pack-year = 20 cigarettes per day for one year).

### PVD events (primary outcome)

The primary endpoint of our study was a new PVD event. During the 1-year follow-up period, the participants underwent a medical consultation within a few days of dizziness or vertigo onset, and a board-certified otorhinolaryngologist at the Oki Clinic diagnosed and reported new PVD events according to the relevant diagnostic guidelines^27^, considering medical history and clinical evidence. Moreover, all diagnoses of PVD were performed by a sonographer (M.W.) blinded to participants smoking status. We classified PVD according to the relevant diagnostic guidelines^27^.

PVD includes VN, BPPV, and MD. We adopted strict diagnostic criteria for these diseases. VN required fulfilment of the following three criteria: (1) a sudden onset of sustained vertigo associated with unidirectional mixed horizontal-torsional spontaneous nystagmus; (2) an absence of cochlear symptom or sign (deafness and tinnitus); and (3) an absence of associated neurological symptoms or signs^28,29^. BPPV required fulfilment of the following four criteria: (1) vertigo associated with a characteristic mixed torsional and vertical nystagmus provoked by the vestibular provocative test including Dix–Hallpike test and roll test; (2) a latency (typically of 1–2 seconds) between the completion of the vestibular provocative test and the onset of vertigo and nystagmus; (3) the paroxysmal nature of the provoked vertigo and nystagmus (i.e. an increase followed by a decline over a period of 10–20 seconds); and (4) fatigue (i.e. a reduction in vertigo and nystagmus when the vestibular provocative test was repeated)^30^. MD required fulfilment of the following three criteria: (1) 2 or more definitive spontaneous episodes of vertigo lasting ≥20 minutes; (2) documented hearing loss on at least one audiological examination; and (3) tinnitus or aural fullness in the treated ear^31^.

### Analysis

We analysed all participants’ data, stratified by sex, because females demonstrate higher PVD incidence than males^32–34^. Data are presented as means ± standard deviations (SD) and population percentages. Continuous variables were compared using t-tests or ANOVA, and categorical variables were compared using chi-square tests. Incidence rates are presented as events per 1000-person years. We calculated the incidence rate for new onset PVDs, according to smoking status (never-smoker, ever-smoker with <30 pack-years and ≥30 pack-years).

We used the Kaplan–Meier method to estimate the cumulative incidence of new PVDs. The Cox proportional-hazard regression model was used to estimate the hazard ratios (HRs) and 95% confidence intervals (CIs) of new PVDs. We used never-smokers as the reference category.

Statistical analyses examined the associations between the incidence of new PVDs and smoking status, after adjusting for confounding factors (age, sex, alcohol consumption status, systolic blood pressure, HbA1c (NGSP), and LDL-C). Statistical significance was defined as *p* < 0.05. All analyses were performed using Stata, version 12.1 (Stata Corp, College Station, TX, USA).

### Data Availability Statement

All available data can be obtained by contacting the corresponding author.

## Results

We examined a total of 393 (133 male, 260 female) participants. The mean participant age at entry was 65.8 ± 8.2 years for all participants, 65.0 ± 9.3 years for male participants and 65.8 ± 7.6 years for female participants. Among all participants, 109/393 (27.7%) were ever-smokers and 284/393 (72.3%) were never-smokers. Among the 133 male participants, there were 90 (67.7%) ever-smokers and 43 (32.3%) never-smokers. Among 260 female participants, there were 19 (7.3%) ever-smokers and 241 (92.7%) never-smokers.

We further divided the ever-smokers into two groups, using a cut-off value of 30 pack-years, based on previous studies. Clinical and biochemical characteristics of the 393 participants stratified by smoking status (never-smoker, ever-smoker with <30 pack-years and ≥30 pack-years) are shown in Table 1. We noted significant differences among the three groups in terms of sex, hypertension, alcohol consumption status, triglyceride levels, total cholesterol, LDL-cholesterol and HDL-cholesterol. Clinical and biochemical characteristics of male and female participants stratified by smoking status (never-smoker, ever-smoker with <30 pack-years and ≥30 pack-years) are shown in Table 2. Among male participants, significant differences in alcohol consumption status and total cholesterol levels were observed among the three groups. Among female participants, significant differences in triglyceride levels, and alcohol consumption status were observed among the three groups.

**Table 1:** Baseline characteristics of the 393 study participants according to smoking status (never-smoker, ever-smoker with <30 and ≥30 pack-year).

Characteristics	never-smoker (n = 284)	ever-smoker (n = 109)		*p* value
		pack-year <30 (n = 39)	pack-year ≥30 (n = 70)	
Age (years)	65.8 ± 8.23	63.6 ± 7.59	65.4 ± 8.53	0.30
Male sex	43 (15.1)	32 (82.1)	58 (82.9)	<0.001
Hypertension	225 (79.2)	36 (92.3)	63 (90)	0.024
Hyperlipidaemia	266 (93.7)	33 (84.6)	65 (92.9)	0.13
Diabetes	32 (11.3)	5 (12.8)	13 (18.6)	0.26
Alcohol consumption status				
Current	30 (10.6)	26 (66.7)	40 (57.1)	<0.001
Former	26 (9.2)	6 (15.4)	9 (12.9)	
Never	228 (80.3)	7 (55.7)	21 (30)	
Blood pressure (mmHg)				
Systolic	132.7 ± 11.3	133.5 ± 9.61	134.0 ± 9.55	0.62
Diastolic	77.0 ± 8.80	77.1 ± 8.14	77.7 ± 7.06	0.82
Triglyceride (mg/dL)	160.8 ± 98.1	165.4 ± 86.0	199.8 ± 134.0	0.020
Cholesterol (mg/dL)				
TC	227.9 ± 37.7	210.3 ± 37.3	214.2 ± 39.7	0.002
LDL-C	137.0 ± 32.3	125.3 ± 33.7	126.3 ± 33.5	0.011
HDL-C	58.7 ± 14.7	52.0 ± 14.4	47.9 ± 12.7	<0.001
HbA1c (NGSP; %)	5.89 ± 0.51	5.92 ± 0.87	5.97 ± 0.64	0.58

Values are represented as mean ± standard deviation or frequency (%).

P values comparing all 3 groups (never-smoker, ever-smoker with <30 and ≥30 pack-years) using ANOVA for continuous variables and chi-square test for categorical variables.

Abbreviations: HDL-C, high-density lipoprotein cholesterol; LDL-C, low-density lipoprotein cholesterol; HbA1c, glycosylated hemoglobin; NGSP, National Glycohemoglobin Standardization Program.

**Table 2:** Baseline characteristics according to smoking status (never-smoker, ever-smoker with <30 and ≥30 pack-years) among male and female participants.

Characteristics	male (n = 133)				female (n = 260)			
	never-smoker (n = 43)	ever-smoker (n = 90)		*p* value	never-smoker (n = 241)	ever-smoker (n = 19)		*p* value
		pack-year <30 (n = 32)	pack-year ≥30 (n = 58)			pack-year <30 (n = 7)	pack-year ≥30 (n = 12)	
Age (years)	65.4 ± 11.2	63.2 ± 7.7	65.7 ± 8.7	0.46	65.9 ± 7.6	65.7 ± 7.5	64.3 ± 7.9	0.77
Hypertension	37 (86.0)	30 (93.8)	52 (89.7)	0.57	188 (78.0)	6 (85.7)	11 (91.7)	0.48
Hyperlipidaemia	41 (95.3)	27 (84.4)	54 (93.1)	0.21	225 (93.4)	6 (85.7)	11 (91.7)	0.72
Diabetes	7 (16.3)	4 (12.5)	11 (19.0)	0.74	25 (10.4)	1 (14.3)	2 (16.7)	0.76
Alcohol consumption Status								
Current	17 (39.5)	23 (71.9)	37 (63.8)	0.005	13 (5.4)	3 (42.9)	3 (25)	<0.001
Former	7 (716.3)	5 (15.6)	5 (8.6)		19 (7.9)	1 (14.3)	4 (33.3)	
Never	19 (44.2)	4 (12.5)	16 (27.6)		209 (86.7)	3 (42.9)	5 (41.7)	
Blood pressure (mmHg)								
Systolic	134.3 ± 9.9	134.6 ± 9.1	134.1 ± 9.7	0.97	132.4 ± 11.5	128.3 ± 11.0	133.8 ± 9.2	0.59
Diastolic	77.8 ± 8.3	77.6 ± 8.2	78.1 ± 6.8	0.96	76.8 ± 8.9	74.4 ± 8.9	75.7 ± 8.1	0.71
Triglycerides (mg/dL)	194.7 ± 105.1	178.5 ± 86.6	195.1 ± 128.5	0.77	154.8 ± 95.7	105.3 ± 53.9	222.6 ± 162.4	0.027
Cholesterol (mg/dL)								
TC	223.0 ± 29.0	204.9 ± 33.0	209.5 ± 37.6	0.049	228.8 ± 39.1	235.1 ± 47.8	237.1 ± 43.1	0.72
LDL-C	133.4 ± 27.1	119.7 ± 30.8	124.2 ± 34.7	0.15	137.7 ± 33.2	151.1 ± 36.9	136.3 ± 26.0	0.56
HDL-C	50.6 ± 13.2	49.6 ± 13.4	46.2 ± 12.1	0.20	60.2 ± 14.5	63 ± 14.7	56.2 ± 12.8	0.55
HbA1c (NGSP; %)	5.90 ± 0.54	5.91 ± 0.86	5.98 ± 0.65	0.83	5.89 ± 0.51	5.93 ± 1.02	5.91 ± 0.61	0.96

Values are represented as mean ± standard deviation or frequency (%).

P values comparing all 3 groups (never-smoker, ever-smoker with <30 and ≥30 pack-years) using ANOVA for continuous variables and chi-square test for categorical variables.

Abbreviations: HDL-C, high-density lipoprotein cholesterol; LDL-C, low-density lipoprotein cholesterol; HbA1c, glycosylated hemoglobin; NGSP, National Glycohemoglobin Standardization Program.

Overall, there were 76 new PVD events (61 VN, 12 BPPV, and 3 MD) during 663.8 person-years of follow-up. Among male participants, there were 219.6 person-years of follow-up, and 19 new PVD events (15 VN, 3 BPPV, and 1 MD). Among female participants, there were 444.3 person-years of follow-up, and 57 new PVD events (46 VN, 9 BPPV, and 2 MD). The incidence rate of the PVD endpoint was 86.5 (95% confidence interval: 52.8–131.6) per 1,000 person-years among male participants and 128.3 (95% CI confidence interval: 98.7–163.1) among female participants. There was no significant difference in PVD incidence between male and female participants (*p* = 0.070).

**Figure 1:**
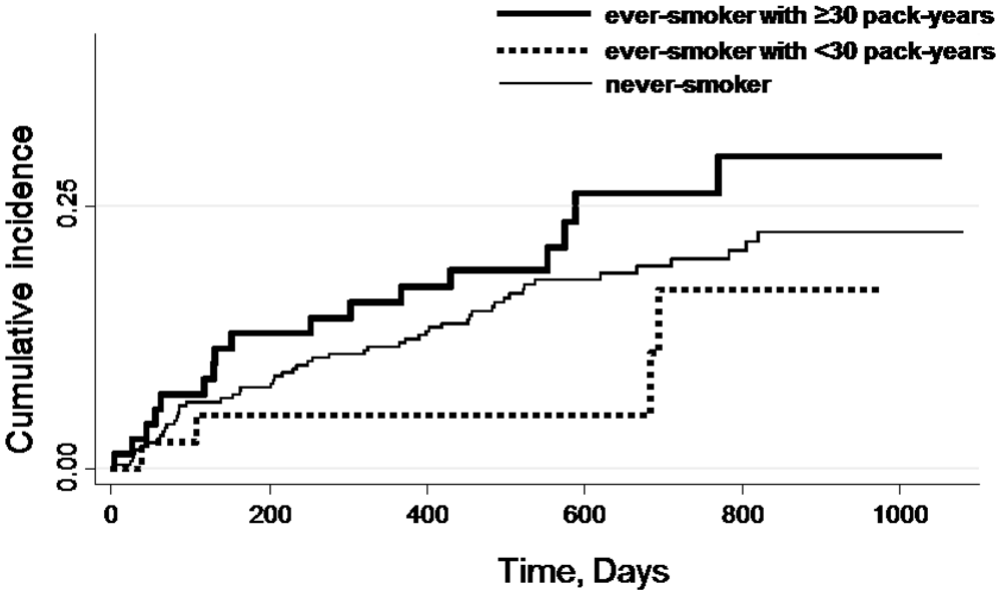
Unadjusted cumulative incidence for the endpoints of peripheral vestibular disorder (PVD), according to smoking status (never-smoker, ever-smoker with <30 and ≥30 pack-years). The number (%) of new events of peripheral vestibular disorder (PVD) was 55 (19.4%) among never-smokers, 4 (10.3%) among ever-smokers with <30 pack-years and 17 (24.3%) among ever-smokers with ≥30 pack-years.

**Figure 2:**
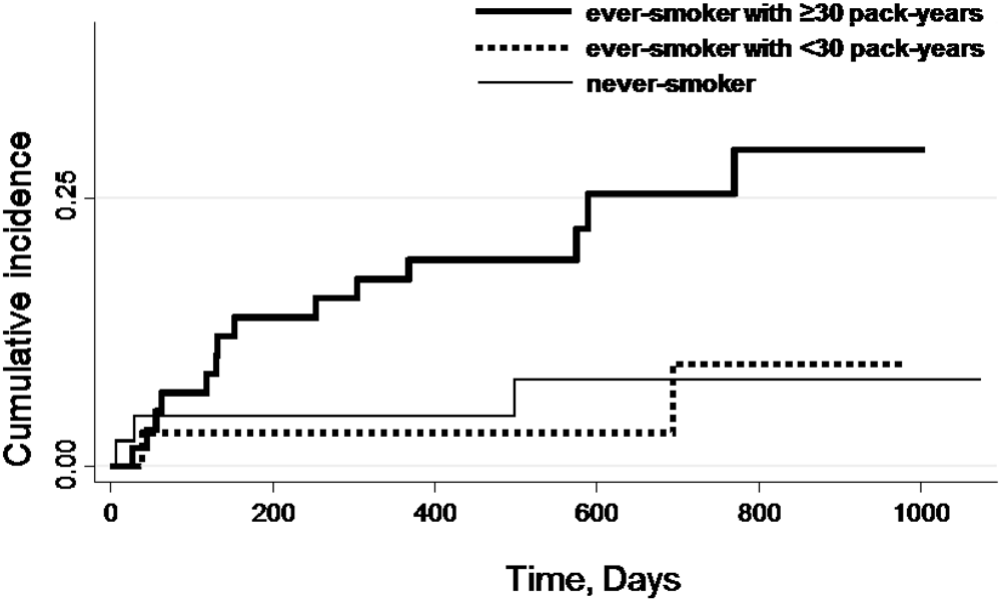
Unadjusted cumulative incidence for the endpoints of peripheral vestibular disorder (PVD), according to smoking status (never-smoker, ever-smoker with <30 and ≥30 pack-years) among 133 male participants. The number (%) of new events of peripheral vestibular disorder (PVD) was 3 (6.98%) among never-smokers, 2 (6.25%) among ever-smokers with <30 pack-years and 14 (24.1%) among ever-smokers with ≥30 pack-years.

**Figure 3:**
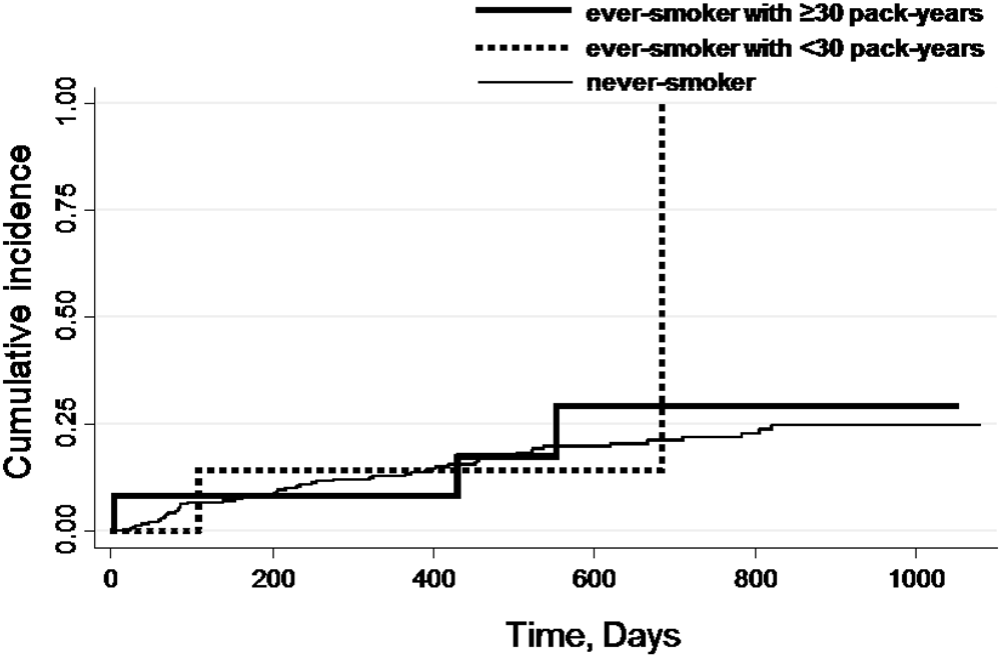
Unadjusted cumulative incidence for the endpoints of peripheral vestibular disorder (PVD), according to smoking status (never-smoker, ever-smoker with <30 and ≥30 pack-years) among 260 female participants. The number (%) of new events of peripheral vestibular disorder (PVD) was 52 (21.6%) among never-smokers, 2 (28.6%) among ever-smoker with <30 pack-years and 3 (25%) among ever-smokers with ≥30 pack-years.

The crude incidence of new PVD events according to smoking status (never-smoker, ever-smoker with <30 pack-years and ≥30 pack-years) is presented in Fig. 1. The number (%) of new PVD events was 55 (19.4%) among never-smokers, 4 (10.3%) among ever-smokers with <30 pack-years and 17 (24.3%) among ever-smokers with ≥30 pack-years. The incidence rate of the PVD endpoint was 112.8 (95% CI: 86.0–144.2) per 1,000 person-years among never-smokers, 58.4 (95% CI: 16.3–143.8) among ever-smokers with <30 pack-years, and 157.6 (95% CI: 94.5–240.0) among ever-smokers with ≥30 pack-years. Figures 2 and 3 show the cumulative incidence of the new PVD endpoint, according to smoking status, among male and female participants. The number of new PVD events and the PVD incidence rate, according to each smoking status category are shown in Table 3.

**Table 3:** Cox proportional-hazard analyses for the endpoints of peripheral vestibular disorder according to smoking status (never-smoker, ever-smoker with <30 and ≥30 pack-years).

	Smoking status	No. of subjects	No. of Events	Crude incidence (1,000 person-years)	Unadjusted		Adjusted for age, sex and other risk factors^*^	
					HR (95% CI)	*p* value	HR (95% CI)	*p* value
All	never	284	55	112.8 (86.0–144.2)	1.00 (reference)		1.00 (reference)	
	ever pack-year <30	39	4	58.4 (16.3–143.8)	0.51 (0.18–1.40)	0.19	1.11 (0.35–3.55)	0.86
	ever pack-year ≥30	70	17	157.6 (94.5–240.0)	1.36 (0.79–2.34)	0.27	2.70 (1.32–5.53)	0.006
Male	never	43	3	41.4 (8.68–117.0)	1.00 (reference)		1.00 (reference)	
	ever pack-year <30	32	2	34.0 (4.13–117.1)	0.81 (0.14–4.88)	0.82	0.91 (0.14–5.87)	0.93
	ever pack-year ≥30	58	14	158.4 (89.8–252.5)	3.61 (1.04–12.58)	0.043	4.41 (1.19–16.4)	0.027
Female	never	241	52	125.3 (95.0–161.1)	1.00 (reference)		1.00 (reference)	
	ever pack-year <30	7	2	206.2 (25.2–556.1)	1.47 (0.36–6.03)	0.60	3.47 (0.78–15.5)	0.10
	ever pack-year ≥30	12	3	154.0 (33.8–395.8)	1.22 (0.38–3.90)	0.74	1.68 (0.52–5.49)	0.39

^*^Adjusted by age, alcohol consumption status, systolic blood pressure, LDL-C, and HbA1c (NGSP) among males and females. (Adjusted by age, sex, alcohol consumption status, systolic blood pressure, LDL-C, and HbA1c (NGSP) among all 393 participants)

Abbreviations: H. R., hazard ratio; CI, confidence interval; LDL-C, low-density lipoprotein cholesterol; HbA1c, glycosylated hemoglobin;

NGSP, National Glycohemoglobin Standardization Program.

Compared to never-smokers, the crude HR was 1.03 (95% CI: 0.62–1.70) for ever-smokers (all participants), 2.53 (95% CI: 0.74–8.69) for male ever-smokers and 1.31 (95% CI: 0.52–3.27) for female ever-smokers. Compared to never-smokers, the HR adjusted for sex, age, alcohol consumption status, systolic blood pressure, serum LDL-C, and HbA1c (NGSP) was 2.22 (95% CI: 1.10–4.49, *p* = 0.027) for ever-smokers (all participants), 3.13 (95% CI: 0.84-11.6, p = 0.088) for male ever-smokers, and 2.10 (95% CI: 0.81-5.43, *p* = 0.13) for female ever-smokers.

Crude and adjusted HRs examining the association between smoking status (never-smoker, ever-smoker with <30 pack-years and ≥30 pack-years**)** and new onset PVD are shown in Table 3. Compared to never-smokers, the HR, adjusted for sex, age, and other risk factors, was 1.11 (95% CI: 0.35-3.55, *p* = 0.86) for ever-smokers with <30 pack-years and 2.70 (95% CI: 1.32-5.53, *p* = 0.006) for ever-smokers with ≥30 pack-years. Compared to never-smokers, HRs adjusted for age and other risk factors were: 0.91 (95% CI: 0.14-5.87, *p* = 0.93) for male ever-smokers with <30 pack-years, 4.41 (95% CI: 1.19-16.4, *p* = 0.027) for male ever-smokers with ≥30 pack-years, 3.47 (95% CI: 0.78-15.5, *p* = 0.10) for female ever-smokers with <30 pack-years, and 1.68 (95% CI: 0.52-5.49, *p* = 0.39) for female ever-smokers with ≥30 pack-years.

## Discussion

Following a review of the medical literature, to the best of our knowledge, this is the first study to examine associations between new onset PVD events and smoking. There was a statistically significant association between smoking and new PVD events. Specifically, among male participants, there was a strong and significant association between a ≥30 pack-year smoking history, and PVD outcomes.

The prevalence of ever-smoking (current or past smoking) was higher among male than female participants. The 2015 Japan National Health and Nutrition Survey^35^, reported that 40.4% of 60–69 year-old males, and 9.5% of 60–69 year-old females were ever-smokers. The proportion of ever-smokers and never-smokers in our study, stratified by sex, was comparable to the results of prior studies^35^.

The incidence of PVD was 86.5 (95% confidence interval: 52.8–131.6) per 1,000 person-years among male participants, and 128.3 (95% confidence interval: 98.7–163.1) per 1,000 person-years among female participants. The incidence of PVD among female participants was approximately two-fold higher than that among male participants, although this difference was not significant (*p* = 0.070). This finding is comparable to the results of many previous studies, showing that the PVD is more common in female^32–34^. Several recent studies reported an association between BPPV, a major cause of PVD, and osteoporosis^36,37^. It is well known that osteoporosis is more common in elderly females than in elderly males^38,39^. There may be a relevant connection between PVD, including BPPV, and osteoporosis; this may be reason that this gender disparity exists.

Previous studies have reported an increased risk for the following conditions in participants with a smoking consumption of over 30 pack-years: lung cancer^23^, chronic obstructive pulmonary disease^25^, myocardial infarction,^25^, and stroke^26^. In this study, we showed that ever-smokers, particularly male smokers with over 30 pack-years, have an increased risk of developing new PVD events. Previous studies found an association between sensorineural hearing impairment and smoking^5–8^. Associations between sensorineural hearing impairment and endothelial dysfunction and vasospasm have also been reported^40–42^. PVD and hearing impairment are major inner ear diseases. Impaired vasodilatation, due to endothelial dysfunction and vasospasm that result from smoking, may cause hearing impairment and new PVD events^40–42^.

Sensorineural hearing impairment is associated with endothelial dysfunction, reduced flow-mediated dilation (FMD), and reduced number of endothelial progenitor cells (EPCs)^43,44^. Endothelial dysfunction is a well-known and established primary smoking effect. Cigarette smoking induces endothelial, inflammatory, and haemostatic markers, such as elevated white blood cell counts^45–47^, cytokines^46–48^, reactive oxygen species (ROS), cyclooxygenase-2 (COX-2)^48^, and increased lipid peroxidation levels^49–51^. These changes may be associated with dose-related, and potentially reversible, impairment of endothelium-dependent dilation^52,53^. Arterial spasms are a reversible form of arterial dysfunction, induced by cigarette smoking. Cigarette smoking increases the risk of coronary spastic angina and acute coronary syndrome^54,55^. A previous study, using multiple regression analysis, revealed that smoking predicted coronary spastic angina (*p* = 0.009)^54^. Another study reported that the highest prevalence of cigarette smoking was found in participants with spastic acute coronary syndrome (*p* = 0.031)^55^. Smoking-induced changes cause transient bloodstream disruption to the labyrinthine artery, a feeding artery to the inner ear, potentially leading to new PVD events. Future studies are required to determine the underlying pathological mechanisms of new PVD onset.

This study has 3 notable strengths. First, the follow-up rate was 100%. This enabled accurate detection of new PVD events. Second, a board-certified otorhinolaryngologist examined participants who complained of dizziness/vertigo in our clinic, allowing accurate detection of PVD. Third, there were no missing data or confounding factors. After adjusting for assumable confounding factors, there was little statistical deflection. Therefore, we estimate that minimal patient selection bias affected the results of this study. Conversely, the present study has several limitations. First, an underreporting bias and a recall bias exist because smoking behaviour were measured by self-report (assessment)^56,57^. Second, our statistical analyses were adjusted for several known confounding factors, although we were unable to account for other, unknown, factors. Third, our sample size was small, particularly for participants with <30 pack-years. There was no statistically significant difference between male ever-smoker with <30 pack-years and the outcome. It is possible that the statistical results may have differed if sample size was larger. Forth, this study involved only one primary care clinic in Japan, and all participants were Japanese. Therefore, these results may not be generalizable to other primary care clinics in Japan or to other countries. Future studies are required to assess the external validity of our findings, by evaluating participants in multicentre and multinational studies.

## Conclusion

In the present study, we revealed that smoking is associated with new PVD events. Specifically, among male participants, smoking consumption of >30 pack-years was strongly associated with an increased risk of new PVD events. This study suggests that smoking habits contribute to cancer risk, cardiovascular disease risk, and PVD (dizziness) in patients with hypertension, dyslipidaemia, and diabetes mellitus. We expect these findings may lead patients with PVD to pursue more aggressive smoking cessation.
